# Fate Decisions of Chicken Primordial Germ Cells (PGCs): Development, Integrity, Sex Determination, and Self-Renewal Mechanisms

**DOI:** 10.3390/genes14030612

**Published:** 2023-02-28

**Authors:** Kennosuke Ichikawa, Hiroyuki Horiuchi

**Affiliations:** 1 Genome Editing Innovation Center, Hiroshima University, 3-10-23 Kagamiyama, Higashi-Hiroshima 739-0046, Hiroshima, Japan; 2 Graduate School of Integrated Sciences for Life, Hiroshima University, 1-4-4 Kagamiyama, Higashi-Hiroshima 739-8528, Hiroshima, Japan

**Keywords:** avian species, chicken, primordial germ cells, fate decision, development, integrity, sex determination, self-renewal, genome editing

## Abstract

Primordial germ cells (PGCs) are precursor cells of sperm and eggs. The fate decisions of chicken PGCs in terms of their development, integrity, and sex determination have unique features, thereby providing insights into evolutionary developmental biology. Additionally, fate decisions in the context of a self-renewal mechanism have been applied to establish culture protocols for chicken PGCs, enabling the production of genome-edited chickens and the conservation of genetic resources. Thus, studies on the fate decisions of chicken PGCs have significantly contributed to both academic and industrial development. Furthermore, studies on fate decisions have rapidly advanced owing to the recent development of essential research technologies, such as genome editing and RNA sequencing. Here, we reviewed the status of fate decisions of chicken PGCs and provided insight into other important research issues that require attention.

## 1. Introduction

The chicken (*Gallus gallus*) is a valuable species in terms of protein resources. Additionally, chickens have been used as a model organism for amniotes to elucidate embryonic developmental mechanisms [1]. Furthermore, chickens have been used as avian models in various areas of biology, thereby contributing to the understanding of vertebrate evolution. Thus, research using chickens greatly enhances both industrial development and developmental and evolutionary biology.

Primordial germ cells (PGCs) are the precursor cells of sperm and eggs. PGCs are the only cell lineage that can transmit genetic information to the next generation. Thus, elucidating the fate decision of PGCs, namely the mechanisms by which they develop, maintain integrity, differentiate into gametes of optimal sexes, and control their self-renewal, is a valuable research subject. Avian PGCs have characteristic developmental features, such as migration into the gonads using the vascular system [2]. Chicken PGCs have also shown a unique sex determination mechanism in which PGC-intrinsic factors may occur in a cell-autonomous manner [3,4,5]. In addition, several studies have revealed the self-renewal mechanism of chicken PGCs, resulting in the establishment of stable culture protocols for chicken PGCs [6,7]. Currently, culturing PGCs is a fundamental technique to establish genome-edited chickens and conserve avian genetic resources so that objective offspring can be produced via germline chimeras transplanted from cultured PGCs and directly incubated [8,9] or incubated with an ex ovo culture system [10]. Therefore, studies on the fate decisions of avian PGCs have demonstrated their unique features and have aided the development of avian biotechnologies.

With the development of essential technologies such as genome editing and RNA sequencing (RNA-seq) analysis, studies on chicken PGCs have shown rapid advancement over the last ten years. For example, the clustered regularly interspaced short palindromic repeats/CRISPR-associated protein (CRISPR/Cas) system [11] has been used not only to produce genome-edited chickens [12], but also to conduct functional analysis of PGC-intrinsic factors via application of this system to cultured PGCs [13]. Alternatively, RNA-seq technology enables us to predict the fate decision of chicken PGCs even at a single-cell resolution level [14,15]. Here, we review the fate decisions of avian PGCs, mainly focusing on chicken PGCs, with cutting-edge knowledge. Furthermore, we discuss the remaining issues that need to be addressed in future studies.

## 2. Early Development of Chicken PGCs

### 2.1. Origin and Identification of Chicken PGCs

In vertebrates, PGC formation is generally classified into two models: preformation and epigenesis [16]. The preformation model has been observed in zebrafish (*Danio rerio*) [17] and anuran amphibians (*Xenopus laevis*) [18]. In this model, germplasm, a maternal factor, acts as a determinant of germ cell formation. The germplasm is composed of maternally inherited RNAs and proteins and is partially asymmetrically partitioned to cells during cleavage divisions and development. As a result, PGCs arise from the partitioned cells. This mechanism is conserved in several non-vertebrate species, including *Drosophila melanogaster* [19] and *Caenorhabditis elegans* [20]. In contrast, the epigenesis model has been shown in urodele amphibians (*Ambystoma mexicanum*) [21] and mammals (*Mus musculus*) [22]. In these species, PGCs are induced in somatic cells via “epigenetic” regulation during embryonic development; thus, the germplasm is absent.

Several studies have been conducted to determine the origins of avian PGCs. In particular, studies focusing on the *vasa* gene have strongly supported the preformation model for avian PGC formation. VASA is a germ-cell-specific RNA helicase that is localized in germ cells as a germplasm component across various species, such as *X. laevis* and *D. melanogaster.* Tsunekawa et al. demonstrated the expression patterns of the chicken vasa homolog (CVH) [23]. The CVH is localized in cleavage furrows and asymmetrically distributed to limited cells. Additionally, the CVH is colocalized with the mitochondrial cloud, corresponding to the germplasm feature in *D. melanogaster.* Recent functional analyses have shown that the CVH significantly contributes to germ cell development in both males and females [24,25]. These studies suggest that chickens possess germplasm-like features and follow the preformation model for PGC formation.

Previous studies targeting deletion in the azoospermia-like (DAZL) protein also support the preformation model in chickens. DAZL is a germ-cell-specific RNA-binding protein localized in the germplasm in some vertebrates [26,27]. In chickens, DAZL is also localized in cleavage furrows as well as the CVH and is specifically expressed in germ cells [28]. Additionally, knockdown of *DAZL* in chicken PGCs causes apoptosis and aberrant expression patterns of germ-cell-characteristic genes, albeit under culture conditions [28]. Recently, whole transcriptome analysis predicted that chicken *DAZL* was co-expressed with its potential interacting genes according to zygotic genome activation, suggesting its central role in germ-cell specification [29].

Therefore, nowadays, avian germ cells are thought to be specified by the preformation model; however, the possibility that avian PGCs are formed via epigenetic regulation cannot be excluded [30]. Thus, while several studies have supported the preformation model in avian PGC formation, the origin of PGCs remains unknown.

### 2.2. Migration of Chicken PGCs into Embryonic Gonads

**Figure 1:**
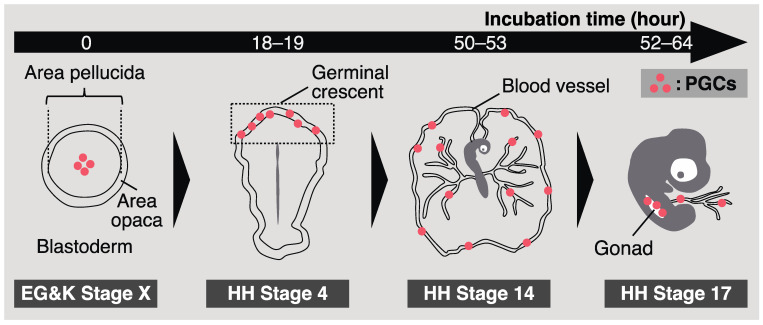
A schematic illustration of chicken primordial germ cell (PGC) development.

A chick embryo in an egg incubates for 0 h, corresponding to Eyal-Giladi and Kochav (EG & K) stage X, and consists of approximately 60,000 cells [31]. At this embryonic stage, approximately 30 CVH-positive cells, namely the origin of PGCs, are scattered at the center of the area pellucida in the blastoderm (Figure 1) [23]. The CVH-positive cells then start to migrate into the germinal crescent, an anterior extraembryonic region. At Hamburger–Hamilton (HH) stage 4 (chick embryos in eggs after 18–19 h of incubation), PGCs accumulated at a high density in the germinal crescent (Figure 1) [32]. Previously, it was thought that PGCs passively translocate into the germinal crescent via the morphogenetic movement of hypoblasts [33]. However, Kang et al. demonstrated that chick fibroblasts exogenously transplanted into the subgerminal cavity of the recipient could not settle in the germinal crescent, whereas transplanted PGCs could [34]. This indicates that PGC-intrinsic factors are also related to this migration. Recently, Huss et al. revealed that quail (*Coturnix japonica*) PGCs contribute to the extracellular matrix in the germinal crescent [35]. Although the molecular mechanism of PGC migration remains unclear, these previous studies showed their “active” role.

After the settlement of PGCs into the germinal crescent, PGCs begin to migrate to the gonads. In many vertebrates, such as mice and zebrafish, PGCs migrate into gonads via “amoeboid migration” [36]. However, in birds (and reptiles), PGCs use the vascular system to migrate into the gonads. Several studies have attempted to elucidate this unique migration system in avian PGCs.

Around HH stage 6 (chick embryos in eggs incubated for 23–25 h), aggregations of endothelial cell progenitors and blood cell progenitors, called blood islands, appear in the extraembryonic mesoderm [37]. Murai et al. demonstrated that more than 60% of quail PGCs in an embryo were enveloped by differentiating endothelial cells forming blood islands in the germinal crescent [38]. Then, the PGCs flowed along with the heartbeat at HH stage 12 (after 48–49 h of incubation). This indicates that most avian PGCs were passively translocated into the vascular system.

Once avian PGCs are translocated into the blood vessels, they start circulating. The concentration of chicken PGCs in the bloodstream reaches a peak at HH stage 14 (after 50–53 h of incubation), and these settle in gonads from HH stage 15 (after 50–55 h of incubation) to HH stage 17 (after 52–64 h of incubation) (Figure 1) [39,40]. Recent studies have demonstrated the molecular mechanisms involved in gonadal migration of avian PGCs. The role of the interaction between chemotactic molecular stromal cell-derived factor 1 (SDF1) and its receptor C-X-C chemokine receptor type 4 (CXCR4) is a well-known system that directs PGCs to the gonads in vertebrates, whose PGCs utilize amoeboid migration [41,42,43,44,45]. In chickens, the expression of CXCR4 has also been observed in PGCs [46], and PGCs are attracted to ectopically expressed SDF1s [47]. Furthermore, the transplantation of *CXCR4* knockout (KO) PGCs into recipient embryos resulted in a reduction in their capacity to migrate into gonads, suggesting a critical role of the CXCR4–SDF1 interaction in this migration [13]. In contrast, recent studies have proposed other factors for gonadal migration of avian PGCs. Saito et al. showed that circulating avian PGCs were stiffer than blood cells; thus, PGCs were efficiently occluded at the vascular plexus near presumptive gonads, resulting in their homing to developing gonads [48]. Huang et al. proposed that platelet-derived growth factor signaling could be involved in the migration of avian PGCs into gonads using RNA-seq analysis [49]. These molecular studies have advanced our understanding of how avian PGCs migrate to gonads. Notably, previous research focusing on the migration of avian PGCs has been conducted mainly using chickens and quails. Thus, whether the molecular mechanism of this migration is conserved across avian species remains unknown.

## 3. Integrity of Chicken PGCs

### 3.1. Epigenetic Regulation

Epigenetic regulation is essential for PGCs to maintain their properties for germinal transmission, namely, establishing their integrity. In mice, PGCs undergo genome-wide epigenetic reprogramming between embryonic day (E) 8.5 and E13.5, and these embryonic stages correspond to the migration and colonization of PGCs to the gonads [50]. Epigenetic reprogramming is required to establish germ cell specification and erase somatic epigenetic memory [51]. The importance of epigenetic modifications in the establishment of PGC integrity has also been observed across species [52].

Several studies have been conducted to reveal epigenetic modifications of chicken PGCs. Yu et al. demonstrated that chicken PGCs undergo global DNA demethylation via ten-eleven translocation 1 during HH stage 21 (after 3.5 d of incubation) to HH stage 28 (after 5.5 d of incubation) [53]. These embryonic stages correspond to states in which chicken PGCs migrate to the gonads and form colonies within the gonads. Rengaraj et al. investigated the expression patterns of the *DNA methyltransferase* (DNMT) families *DNMT1*, *DNMT 3 α (DNMT3A)*, and *DNMT 3 β (DNMT3B)* in chicken PGCs during embryogenesis and suggested that DNMT3B-dependent de novo DNA methylation occurred after PGCs settled into the gonads [54]. Jang et al. showed the characteristic DNA methylation patterns of gonadal PGCs by comparing them with those of chicken embryonic fibroblasts [55]. Although the understanding of DNA methylation in PGCs is not yet complete, these analyses are beginning to elucidate the underlying mechanism.

Furthermore, several studies have been reported concerning the elucidation of histone modification in avian PGCs. In mice, after the transient loss of histone modifications during epigenetic reprogramming, PGCs regain both histone H3 lysine 9 (H3K9) and H3K27 trimethylation (me3) around E12.5 [56]. However, histone modification of chicken PGCs was based on H3K9me3 rather than H3K27me3, suggesting the existence of avian-specific epigenetic regulation in PGCs [57]. For other modifications, H3K4me2 activates signaling pathways essential for avian PGC formation, such as bone morphogenetic protein 4 (BMP4) signaling [58]. Additionally, H3K9 acetylation (H3K9ac) contributes to maintaining the integrity of avian PGCs via the regulation of *NANOG*, a key transcription factor for germ cell development [59] (described below). These analyses have revealed histone modifications in avian PGCs, including avian-specific H3K9me3-dominant gene expression regulation.

### 3.2. Key Molecules for the Integrity of Avian PGC

Key molecules for the integrity of PGCs, including transcription factors, are well-conserved in vertebrates. Recently, several researchers conducted functional analyses of these molecules in avian PGCs. Interestingly, these key molecules exhibited bird-like features. In this section, we describe the functions and characteristics of the molecules involved in the integrity of chicken PGCs, along with their differences from those in other model organisms.

#### 3.2.1. PRDM14 and BLIMP1

*PR-domain-containing protein 14* (*Prdm14*) and *B lymphocyte-induced maturation protein 1* (*Blimp1*) are transcription factors essential for the specification of PGCs in mammals. In mice, PRDM14 contributes to epigenetic reprogramming, the reacquisition of potential pluripotency, and PGC-specific gene expression [60,61]. In contrast, BLIMP1 represses somatic gene expression in PGCs [62]. In chickens, both PRDM14 and BLIMP1 facilitate PGC development. Okuzaki et al. demonstrated that *PRDM14* and *BLIMP1* were expressed in blastodermal cells and PGCs, and the knockdown of each gene in chicken embryos decreased the number of PGCs [63]. Interestingly, the lack of *PRDM14* causes embryonic lethality in chickens, even though *Prdm14* KO mice are viable [64]. This implies avian-specific functions of PRDM14 during early embryogenesis.

#### 3.2.2. BMP4

Bone morphogenetic protein 4 (BMP4) directly induces the formation of mouse PGCs in an epigenetic model. In mice, BMP4 is emitted into the extra-embryonic ectoderm and induces *Prdm14* and *Blimp1* expression, resulting in the PGC fate [65,66]. In this fate decision, Wnt signaling is also involved in terms of inducing the *Blimp1* expression [66,67]. In chickens, *BMP4* is also essential for the integrity of PGCs. While the nucleotide sequence in the promoter region of chicken *BMP4* is poorly conserved with mammalian *Bmp4*, the protein sequences in the coding region of BMP4 are highly conserved in birds and mammals [30]. Using chicken embryonic stem cells (ESCs) [68,69], Zuo et al. demonstrated that BMP4 contributes to the induction of PGC formation from the ESCs [70,71,72]. However, the molecular mechanisms underlying this induction may differ between chickens and mammals. In chickens, Wnt signaling activated by BMP4 directly regulates *lin-28 homolog A* (*LIN28A*), a significant factor for PGC formation [73]; this direct interaction between Wnt signaling and *LIN28A* has not yet been observed in the fate decisions of mammalian PGCs [72]. Additionally, a recent study showed that a long non-coding RNA, LncBMP4, has similar functions to chicken BMP4 and contributes to the fate decisions of PGCs [74]. Overall, studies on chicken BMP4 have contributed to both the elucidation of the fate decisions of chicken PGCs and the improvement in methods to induce PGCs from avian pluripotent stem cells.

#### 3.2.3. NANOG

NANOG is a transcription factor involved in maintaining pluripotency in ESCs [75,76]. Additionally, NANOG plays a crucial role in the specification of mammalian PGCs via their epigenetic modification [77,78]. In chicken ESCs, NANOG also regulates pluripotency [79]. Because chicken NANOG can function as an alternative factor to mouse NANOG and contribute to the pluripotency of mouse cells [80], its function is conserved between mammals and birds, at least in terms of stem cell pluripotency. Interestingly, the oligomerization mechanism of NANOG proteins differs between mammals and chickens [81]. In addition, NANOG is essential for the somatic reprogramming of chicken and duck (*Anas platyrhynchos*) fibroblasts [82]. In chicken PGCs, *NANOG* expression has been observed, and its function in the self-renewal of PGCs has been suggested [63,83]. However, its expression level is downregulated during the migration of PGCs to the gonads, differing from the expression patterns observed in mouse PGCs [84]. Recent studies have revealed the expression regulation mechanisms of *NANOG* in chicken PGCs. Deacetylation of H3K9ac and several transcription factors, such as tumor protein p53 (TP53), POU domain class 5 transcription factor 3 (POU5F3), and SRY-box 2 (SOX2), control the expression of *NANOG* [59,85]. Nevertheless, alterations in the expression patterns of *NANOG* in chicken PGCs during embryogenesis remain unclear. Overall, although the function of chicken NANOG is similar to that of mammals, its expression pattern, regulatory mechanism, and oligomerization mechanism are different from those of mouse NANOG.

#### 3.2.4. POU5F3

POU5F1 (also called octamer-binding transcription factor 4 (OCT4)) is an essential transcription factor for maintaining the pluripotency of ESCs and specification of the inner cell mass in mice [86,87,88]. Additionally, POU5F1 plays a critical role in establishing mammalian-induced pluripotent stem cells (iPSCs) [89]. Furthermore, POU5F1 is required for maintaining germ cell lineage in mice [90]. However, a previous study demonstrated that *POU5F1* is evolutionarily lacking in chickens [91], which remained the prevailing theory until Lavial et al. successfully cloned the *POU V* gene as a homologous gene of *Pou5f1* and identified its function in maintaining chicken ESCs [79]. Further studies have proposed that chicken *POU V* establishes synteny with the *POU5F3* gene, which is conserved in several vertebrates, such as marsupials, monotremes, and teleost fishes [92,93,94]. Additionally, Nakanoh et al. recharacterized the nucleotide sequence of chicken *POU V* and showed its similarity with those from *POU5F3* in other species [95]. In terms of maintaining the pluripotency of ESCs, the degree of functional conservation between POU5F1 and POU5F3 is species-dependent [94]. Therefore, it is thought that the relationship between chicken *POU V* (*POU5F3*) and mammalian *Pou5f1* is a paralog rather than an ortholog.

In chicken PGCs, *POU5F3* is expressed along with *NANOG* and *SOX2* and may form a transcriptional network [84,96]. A recent study demonstrated that POU5F3 regulates globally active chromatin modification [97]. In addition, the knockdown of *POU5F3* in chicken PGCs resulted in the severe impairment of their gonadal colonization [97]. These studies suggest an essential role of POU5F3 in chicken PGCs.

#### 3.2.5. DND1

DND microRNA-mediated repression inhibitor 1 (DND1), an RNA-binding protein, maintains the fate of PGCs in vertebrates. In mice, DND1 inhibits apoptosis of germ cells by destabilizing target mRNAs [98,99]. In zebrafish, DND1 also regulates apoptosis [100]. Recently, Gross-Thebing et al. clarified that zebrafish DND1 mainly controls somatic differentiation of PGCs, and the lack of *DND1* causes apoptosis, depending on the case [101]. In chickens, the *DND1* homolog was cloned, and its germ-cell-specific expression pattern was characterized [102,103]. However, the function of the chicken DND1 homolog remains unclear. To reveal the molecular mechanisms that suppress somatic differentiation in chicken PGCs, functional analyses of the chicken DND1 homolog are needed to provide new insights.

#### 3.2.6. Non-Coding RNA

Non-coding RNAs (ncRNAs) play key roles in germ cell development. PIWI-interacting RNAs (piRNAs) are a class of non-coding RNAs involved in germ cell development. piRNAs are non-coding RNAs of approximately 26–31 nucleotides in length strongly expressed in the gonads and control germ cell development by repressing transposons to maintain genomic integrity [104,105,106,107]. In chickens, piRNAs specifically expressed in germ cells have been identified by RNA-seq analyses [108,109]. Repression of piRNA pathways in chicken PGCs results in increased DNA double-strand breaks, indicating that piRNAs play a critical role in the integrity of PGCs [108].

Critical roles of other classes of non-coding RNAs, such as microRNAs (miRNAs) and lncRNAs, have also been reported with regard to the integrity of chicken PGCs. miRNAs include 18–23 nucleotides related to the post-transcriptional regulation of target mRNAs. Lee et al. identified miRNAs that are expressed explicitly in chicken PGCs [110]. They demonstrated that miR-181a* downregulates the expression of *nuclear receptor subfamily 6 group A member 1* (*NR6A1*), and *homeobox A1* (*HOXA1*), suggesting that miR-181a* regulates inappropriate meiosis and somatic differentiation of chicken PGCs in different pathways [110]. Other studies have also identified and characterized miRNAs involved in maintaining the integrity of chicken PGCs, such as the regulation of DNA methylation [54], glucose metabolism [111], and apoptosis [112]. In contrast, lncRNAs are non-coding RNAs composed of over 200 nucleotides and possess various functions, such as epigenetic, transcriptional, and translational regulation, via lncRNA–RNA or lncRNA–protein interactions [113]. Zuo et al. identified lncRNAs specifically expressed in chicken PGCs and demonstrated that lncRNA PGC transcript-1 (lncPGCAT-1) contributes to the formation of chicken PGCs by regulating *CVH* expression [114]. Other studies have also characterized several lncRNAs related to the development of chicken PGCs [115,116,117]. Overall, previous studies have demonstrated that non-coding RNAs possess various functions and contribute to chicken PGC integrity.

## 4. Sex Determination in Birds

### 4.1. Bird-Characteristic Sex Determination Mechanisms

Birds exhibit unique features of sex determination. The development of the urogenital system in birds resembles that in mammals. In contrast, birds naturally experience sex reversal, indicating the considerable role of sex hormones in avian sex determination, as well as that in fish. Indeed, Elbrecht and Smith experimentally demonstrated that the inhibition of aromatase, an enzyme that converts testosterone to estradiol, confers a male phenotype on genetically female chickens [118]. Nevertheless, naturally occurring gynandromorphous birds indicate that a cell-autonomous manner significantly determines the sexual identity of avian somatic cells [119,120]. Given the above, birds show characteristic features in their sex determination, but their comprehensive mechanisms remain unclear. Elucidating this mechanism is essential to understand vertebrate evolution in terms of sex determination.

In several vertebrates, the master transcription factors for gonadal sex determination, namely the fate decision of the testes or ovaries, have been determined. In mice, the *sex-determining region Y* (*Sry*) [121], which is located on the Y chromosome, acts as the master gene. In teleost fish medaka, a Y-chromosome-linked gene, the *DM domain gene on the Y chromosome* (*DMY*), determines gonadal sex [122]. Furthermore, in *X. laevis*, which uses a ZZ/ZW sex-determining system, the W-chromosome-linked DM domain gene (*DM-W*) was identified as the master gene [123].

**Figure 2:**
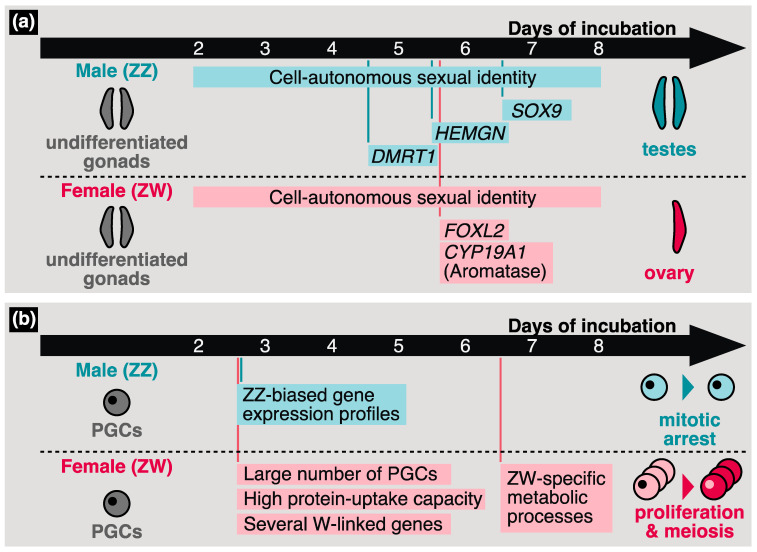
Schematic images of chicken sex determination in undifferentiated gonads (**a**) and primordial germ cells (PGCs) (**b**). The timing of the expression of sex-determination-related genes and the sexual phenotypes observed or predicted in the previous studies are illustrated.

A schematic image of gonadal sex determination in chickens is shown in Figure 2a. Birds also use the ZZ/ZW system; however, leading candidates for W-linked master transcription factors have not yet been identified. In contrast, significant effects of a Z-linked gene, *doublesex and mab-3-related transcription factor 1* (*DMRT1*), for avian gonadal sex determination have been reported. Birds at least partially lack a compensation system for Z-chromosome-linked genes, which corresponds to mammalian X inactivation [124,125]. From HH stage 25 (after 4.5 d of incubation) onwards, higher *DMRT1* expression in male (ZZ) chickens than in female (ZW) chickens has been observed in undifferentiated chicken gonads [126]. Subsequent functional analyses showed that DMRT1 significantly contributes to gonadal masculinization [127,128]. Interestingly, recent studies demonstrated that hetero KO of DMRT1 in ZZ chickens leads to incomplete female morphology, although ovarian development is induced [129,130]. This suggests that cell-autonomous sex identity is essential for sex determination in avian somatic cells.

Other sex-determination-related genes have also been identified in birds. *hemogen* (*HEMGN*) is a chicken-specific gonadal-masculinization-related transcription factor. In mice, *Hemgn* is specifically expressed in hematopoietic tissues and cells [131]. However, in chickens, the upregulation of *HEMGN* is observed in undifferentiated male gonads from HH stage 28 (after 5.5 d of incubation) onwards, and this expression timing suggests that HEMGN operates downstream of DMRT1 [132]. Overexpression of *HEMGN* in ZW chicken embryos induces the expression of *SRY-box 9* (*SOX9*), a testicular marker [132]. The essential role of the *forkhead box L2* (*FOXL2*) gene in gonadal feminization in birds has also been characterized. *FOXL2* expression is increased in undifferentiated ZW gonads from HH stage 28 to 29 (after 5.7 d of incubation) [133]. FOXL2 suppresses the expression of male pathway genes such as *SOX9* and activates ovarian development [134]. Although the expression pattern of *cytochrome P450 family 19 subfamily A member 1* (*CYP19A1*), which encodes for aromatase, resembles that of *FOXL2*, FOXL2 might not directly regulate *CYP19A1* (Aromatase) expression [133,134]. Thereafter, the undifferentiated gonads of the ZZ and ZW embryos develop symmetrically and asymmetrically, respectively, and both the right and left undifferentiated gonads of the ZZ embryo develop functional testes, while only the left gonad becomes a functional ovary in the ZW embryo.

### 4.2. Sexual Identity of Avian PGCs

In several vertebrates, the sex of germ cells is affected by gonadal somatic cells following the gonadal migration of PGCs. However, in birds, several researchers have observed the cell-autonomous sexual identity of PGCs (Figure 2b). First, the number of chicken PGCs migrating to undifferentiated gonads is greater in females than in males [40]. Second, chicken PGCs cultured and harvested from female embryos showed a lower tendency for self-renewal compared to those from male embryos [135]; therefore, the culturing of female chicken PGCs was difficult compared to that of male chicken PGCs. Thus, the culture protocol for chicken PGCs has been optimized for each sex [7]. Third, the capacity for protein uptake differs between male and female PGCs [4]. These reports suggest that the sexual identity of initial PGCs is constructed by PGC-intrinsic factors independent of gonadal sex determination.

In vertebrates, medaka *forkhead box L3* (*FOXL3*) was first identified as a germ-cell-intrinsic sex determination factor. *FOXL3* KO female medaka developed functional sperm in the ovary, indicating that the sex determination mechanism differs between PGCs and gonads [136,137]. Subsequently, in tilapia (*Oreochromis niloticus*), both *FOXL3* and *DMRT1* significantly contribute to the sex determination of PGCs [138]. Recently, the *FOXL3-like gene* was cloned and characterized in chickens [3]. Although the chicken FOXL3-like protein is specifically expressed in female embryonic germ cells, its expression timing is considerably late compared to gonadal sex differentiation, suggesting that the chicken *FOXL3-like gene* may not affect the sexual identity of PGCs [3]. In addition, chicken PGC-intrinsic *DMRT1* may not affect the initial sexual identity of PGCs owing to its expression pattern; sex-biased *DMRT1* expression is temporally observed in female germ cells around HH stage 42 (after 16 d of incubation), which corresponds to the meiotic stage [14]. Taken together, although the chicken *FOXL3-like gene* and *DMRT1* may be essential for germ cell development, the molecular mechanism of initial sex determination in chicken PGCs remains unclear.

Recent studies have shown characteristic avian features in the sex determination of PGCs. Ichikawa et al. conducted RNA-seq analysis using male and female PGCs harvested from embryonic chicken blood at HH stage 17 (after 2.5 d of incubation) and gonads at HH stage 25–26 (after 4.5 d of incubation) and HH stage 30 (after 6.5 d of incubation) [5]. The gene expression profiles obtained in that study suggest that several W-chromosome-linked genes are expressed in a cell-autonomous manner in female PGCs before they settle into the gonads (Figure 2b) [5]. Furthermore, alterations in gene expression profiles were observed during gonadal sex determination in female PGCs prior to that in male PGCs, and female-biased genes were enriched in several metabolic processes (Figure 2b) [5]. In contrast, Rengaraj et al. showed male-biased gene expression in chicken PGCs collected from early embryos around HH stage 17–34 (after 2.5–8 d of incubation) (Figure 2b) [14]. These differences may be due to the methods used to collect the PGCs; while Ichikawa et al. purified the PGCs using FACS targeting stage-specific embryonic antigen-1 (SSEA-1), Rengaraj et al. used DAZL as the marker of PGCs. Although SSEA-1 has been widely used as a PGC marker [46,139,140,141], a recent study demonstrated the existence of SSEA-1-negative PGCs [142]. Note that SSEA-1-positive PGCs, at least, possess germline transmission [141]. In summary, although the timing and molecular mechanisms of sex determination in avian PGCs remain unknown, the gene expression profiles obtained from recent studies can significantly contribute to solving these issues.

Importantly, the biological significance of the initial sexual identity of avian PGCs remains unclear. Previously, it was thought that the sexual identity of PGCs is constructed when the PGCs migrate into the undifferentiated gonads owing to the results of transplantation experiments. While transplantation of the precursor cells of PGCs harvested from the blastodermal stage into recipients of the opposite sex could differentiate into functional gametes [143], the migrating PGCs could barely differentiate those [144,145]. However, a recent study demonstrated that transplanted chicken PGCs circulating through blood vessels could differentiate into gametes of opposite sexes using genetically infertile chickens as recipients [146]. These differences may reflect that initial sexual identity does not completely determine the sexual fate of PGCs.

### 4.3. Sex Differentiation of Avian Germ Cells

Remarkable phenotypic differences between male and female chicken PGCs were observed after gonadal sex determination (Figure 2b). The number of chicken female germ cells is significantly increased from HH stage 35 (after 9 d of incubation) onwards, while male PGCs undergo mitotic arrest until hatching [147,148]. At HH stage 38 (after 12.5 d of incubation), *stimulated by retinoic acid 8* (*STRA8*) is expressed in chicken female germ cells and induced by retinoic acid (RA) [149,150]. Then, the female PGCs undergo meiosis at HH stage 41 (after 15.5 d of incubation). In the early embryonic stages, both major enzymes involved in RA synthesis and RA degradation, retinaldehyde dehydrogenase, type 2 (RALDH2), and cytochrome P450 family 26 subfamily B member 1 (CYP26B1), respectively, are expressed in the gonads of each sex. In females, the expression of *CYB26B1* is downregulated after HH stage 36 (after 10.5 d of incubation), which may be related to female-specific entry into meiosis [149]. Given the above, typical sexual differentiation in chicken germ cells is characterized by differences in proliferation activity and female-specific meiosis.

## 5. Self-Renewal of Chicken PGCs

**Figure 3:**
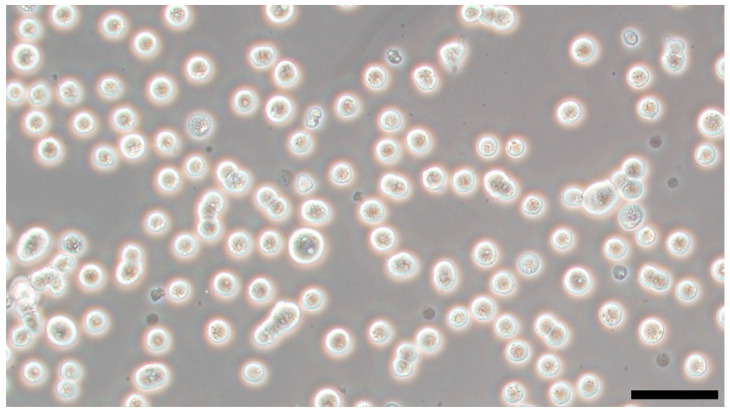
A photograph of cultured chicken primordial germ cells (PGCs). The PGCs were cultured according to a protocol established by Ezaki et al. (2020) [159]. Scale bar = 50 μm.

Among vertebrates, chicken is the only species in which PGCs can be cultured for a long time (Figure 3). Owing to the fact that abundant egg yolk yields surround one-cell fertilized chicken eggs, direct injection of genome editing reagents is difficult. Thus, genome-edited chickens have been produced via gene modification and transplantation of cultured PGCs [151]. Currently, genome-edited chickens are widely used for the study of avian developmental biology [24,64,129,130], production of hypoallergenic eggs [12,152,153], improvement in production efficiency [154], and establishment of bioreactors [155,156]. Furthermore, culture technology can contribute to the conservation of rare avian strains [157]. Therefore, culturing chicken PGCs is an effective technique for fundamental research and industrial applications.

In 2006, van de Lavoir et al. reported a culture protocol for chicken PGCs using the fibroblast growth factor (FGF), stem cell factor (SCF), and mammalian feeder cells, for the first time [6]. Since this achievement, several researchers have attempted to improve their culture efficiency and have revealed essential factors for the self-renewal of cultured PGCs. Optimization of the culture protocol was conducted by Miyahara et al., who demonstrated that the membrane-bound form of the chicken SCF (SCF2) could aid in the proliferation of cultured PGCs [135,158]. Whyte et al. identified the minimal number of signaling pathways required for the self-renewal of cultured PGCs and reconstructed the culture protocol. They showed that FGF2, insulin, and activin significantly contribute to the culture of PGCs, which induce the activation of ERK1/2, Akt, and SMAD3, respectively [7]. In this minimal condition, the SCF is not required. Thus, Whyte et al. hypothesized that insulin might replace SCF function [7]. In addition, several small molecules, such as blebbistatin and CHIR99021, induce the activation of the self-renewal of cultured PGCs by reducing apoptosis [159,160]. Furthermore, Chen et al. established a three-dimensional culture system for chicken PGCs, enabling the efficient culture of chicken PGCs on a large scale [161].

The studies described above played a significant part in the development of a method for culturing chicken PGCs. However, current culture protocols cannot maintain PGCs derived from avian species other than chicken. For example, although researchers have attempted to culture PGCs derived from several avian species, such as quail, duck, and zebra finch (*Taeniopygia guttata*), a stable culture of PGCs, as shown in chicken PGCs, could not be achieved [162,163,164]. Furthermore, current culture protocols have been applied only to certain chicken strains. Woodcock et al. showed that difficulties in culturing chicken PGCs differ among strains [157]. Therefore, to promote avian biotechnology and biodiversity conservation, the current culture protocol for chicken PGCs should be further expanded to include a generic protocol for all avian PGCs.

While recent studies have reported strategies to produce genome-edited birds without cultured PGCs, the value in improving the current culture protocols remains undeniable. For example, a method involving intracytoplasmic injection of sperm has been established in quails, and Mizushima et al. successfully produced genome-edited quails via the direct injection of CRISPR/Cas components into fertilized quail eggs [165,166]. However, this strategy requires advanced technology, and the production of genome-edited birds using this strategy has not yet been achieved in birds other than quail. In addition, methods using adenoviral vectors [167] and sperm [168] as delivery systems to induce CRISPR/Cas components have also been reported. Furthermore, a recent study produced chicken iPSC-derived offspring [169], which could contribute to the production of genome-edited birds without using cultured PGCs. Despite the potential for application in all avian species, the efficiency of producing objective offspring remains low. Therefore, these methods are not widely used. In summary, improvements in the culture protocols of PGCs are needed to further develop avian molecular biology as well as to improve the advanced strategies described above.

## 6. Conclusions

Elucidating the mechanisms of avian PGC fate decisions has long been an essential research topic. Recently, this research subject has received further attention owing to the application of genome editing technology and RNA-seq analysis. Further studies on the early development, integrity, and sex determination of chicken PGCs will provide significant insights into evolutionary developmental biology. Additionally, studies on sex determination using avian PGCs will contribute to the control of the sex of offspring, an essential technology for both efficient poultry production and animal welfare. Moreover, further studies on the self-renewal of chicken PGCs may make it possible for all avian species to be genetically conserved and modified by improving PGC culture protocols. Nonetheless, as many challenges remain unaddressed, the investigation of fate decisions is an essential research subject for future studies.

## Data Availability

Not applicable.
